# Near-Complete Genome Sequences of Vesicular Stomatitis Virus Indiana Laboratory Strains HR and T1026R1 and Plaque Isolates 22-20 and 22-25

**DOI:** 10.1128/MRA.00012-19

**Published:** 2019-04-04

**Authors:** Thomas M. Russell, Evan E. Santo, Lisa Golebiewski, Nathan S. Haseley, Maureen C. Ferran

**Affiliations:** a Thomas H. Gosnell School of Life Sciences, Rochester Institute of Technology, Rochester, New York, USA; KU Leuven

## Abstract

We report four near-complete genome sequences of vesicular stomatitis virus (VSV) Indiana obtained with Sanger and Illumina next-generation sequencing, namely, laboratory strains HR (heat resistant) and T1026R1 and isolates 22-20 and 22-25. Previously, only the M gene of these viruses had been sequenced, and these sequences were not deposited in GenBank.

## ANNOUNCEMENT

Vesicular stomatitis virus (VSV) is a member of the genus *Vesiculovirus* in the family *Rhabdoviridae* and has an ∼11-kb nonsegmented, negative-sense, single-stranded RNA genome that encodes the nucleoprotein (N), matrix (M), glycoprotein (G), large (L), and phosphoprotein (P) proteins (1). We report the near-complete genome sequences of VSV Indiana laboratory strains HR (heat resistant) (2, 3) and T1026R1 (4) and plaque isolates 22-20 and 22-25, derived from Indiana parent strain 22 (03/87-CR-B, number 22), which was originally isolated from an infected cow (5). HR encodes a wild-type (wt) M protein and is therefore a virus that suppresses interferon (IFN) and inhibits host transcription. T1026R1 encodes a M51R mutation in the M protein which abrogates these functions of M (6–8). The M genes of 22-20 and 22-25 were reported to be identical, even though these isolates are discordant for IFN production in chicken embryo cells (9, 10). To determine if another viral component regulates IFN suppression, we sequenced the majority of these four virus genomes.

Virus stocks were propagated in Vero cells at a multiplicity of infection of 0.0001. Once 80 to 90% of the cells exhibited a cytopathic effect, the supernatant was harvested, centrifuged at 4,000 × *g*, aliquoted, and stored at −80°C. At least two virus stocks of each strain were sequenced and found to be identical. Sanger sequencing was performed first, and Illumina sequencing confirmed these results. Sanger and Illumina sequences were aligned for each virus to generate a consensus genome. For Sanger sequencing, the total RNA was extracted from L929 cells at 4 h postinfection (RNAqueous-4-PCR kit, Ambion). Reverse transcription, PCR, product purification, and sequencing were performed as previously described (11). For Illumina MiSeq sequencing, RNA was extracted from 1 ml of virus stock (QIAamp UltraSens virus kit, Qiagen). RNA quality and concentration were determined (Bioanalyzer Agilent RNA 6000 NanoAssay), and a library was constructed (TruSeq Stranded mRNA sample preparation kit, Illumina). The samples were pooled, and the multiplexed samples produced an average of 207,990 paired-end reads per sample (2 × 151 read length). Files were demultiplexed (bcl2fastq version 2.19.0), and adapters and low-quality reads were removed (Trimmomatic version 0.36) (12). Processed, cleaned reads were mapped to the human reference genome (GRCh38+gencode28) with STAR version 2.6.0c (13). Unmapped reads were then assembled *de novo* with SPAdes version 3.11.1 (14).

Sanger and Illumina contigs were aligned to each other and mapped to the NCBI reference sequence (GenBank accession number NC_001560) with CodonCode Aligner (CodonCode Corp.). Open reading frames (ORFs) were predicted with ORF Finder (Web version, NCBI) (15). Genome comparison of HR and T1026R1 confirmed the M51R mutation in the T1026R1 M protein and revealed a novel amino acid substitution in the T1026R1 G protein (S431A). Comparison of 22-25 and 22-20 revealed a previously unidentified D-to-G amino acid substitution at position 52 of the 22-20 M protein (D52G). A single amino acid change was identified in the 22-20 M protein, which suggests that this protein alone suppresses the IFN response. The genomes recovered ranged from 11,115 to 11,143 nucleotides in length, obtaining 99.59 to 99.84% of the genomes relative to the reference sequence. The missing nucleotides are located in noncoding regions at the ends of the genomes.

### Data availability.

Genome sequences of 22-20, 22-25, T1026R21, and HR were deposited in GenBank (accession numbers MH919396, MH919397, MH919398, and MH919399, respectively). Raw Illumina reads were submitted to the Sequence Read Archive (BioProject number PRJNA508804).
